# Supplementary material for: Protocol for the development of a tool (INSPECT-SR) to identify problematic randomised controlled trials in systematic reviews of health interventions
Source: BMJ Open. 2024 Mar 11;14(3):e084164. doi: 10.1136/bmjopen-2024-084164 (PMC10936473; doi:10.1136/bmjopen-2024-084164)
Supplement: Author's manuscript [file bmjopen-2024-084164.draft_revisions.pdf]

BMJ Open is committed to open peer review. As part of this commitment we make the peer review history of every article we publish publicly available.

When an article is published we post the peer reviewers' comments and the authors' responses online. We also post the versions of the paper that were used during peer review. These are the versions that the peer review comments apply to.

The versions of the paper that follow are the versions that were submitted during the peer review process. They are not the versions of record or the final published versions. They should not be cited or distributed as the published version of this manuscript.

BMJ Open is an open access journal and the full, final, typeset and author-corrected version of record of the manuscript is available on our site with no access controls, subscription charges or pay-per-view fees (http://bmjopen.bmj.com).

If you have any questions on BMJ Open's open peer review process please email info.bmjopen@bmj.com

BMJ Open

Protocol for the development of a tool (INSPECT-SR) to identify problematic randomised controlled trials in systematic reviews of health interventions.

Journal:	*BMJ Open*
Manuscript ID	bmjopen-2024-084164
Article Type:	Protocol
Date Submitted by the Author:	11-Jan-2024

Complete List of Authors:	Wilkinson, Jack; Manchester Academic Health Science Centre, Centre for Biostatistics<br>Heal, Calvin; Centre for Biostatistics, University of Manchester, Manchester Academic Health Science Centre; Salford Royal NHS Foundation Trust,<br>Antoniou , George ; Manchester University NHS Foundation Trust, Department of Vascular and Endovascular Surgery; The University of Manchester Faculty of Biology Medicine and Health, Division of Cardiovascular Sciences<br>Flemyng, Ella; Cochrane, Evidence Production and Methods Directorate<br>Alfirevic, Zarko; University of Liverpool, Department of Women's and Children's Health<br>Avenell, Alison; University of Aberdeen, Health Services Research Unit<br>Barbour, Ginny; Medical Journal of Australia<br>Brown, Nicholas; Linnaeus University, Department of Psychology<br>Carlisle, John; Torbay Hospital, Anaesthesia and Critical Care<br>Clarke, Mike; Queen's University Belfast, Northern Ireland Methodology Hub<br>Dicker, Patrick; Royal College of Surgeons in Ireland, Department of Epidemiology and Public Health<br>Dumville, Jo C.; The University of Manchester<br>Grey, Andrew; University of Auckland, Department of Medicine<br>Grohmann, Steph; Cochrane<br>Gurrin, Lyle; University of Melbourne School of Population and Global Health, School of Population and Global Health<br>Hayden, Jill; Dalhousie University, Community Health & Epidemiology<br>Heathers, James; SafeBeat Rx Inc<br>Hunter, Kylie; NHMRC Clinical Trials Centre, University of Sydney<br>Lasserson, Toby; Cochrane, Evidence Production and Methods Directorate<br>Lam, Emily; Independent Lay Member<br>Lensen, Sarah; University of Melbourne, Obstetrics and Gynaecology<br>Li, Tianjing; University of Colorado, Department of Ophthalmology<br>Li, Wentao; Monash University, Department of Obstetrics and Gynecology<br>Loder, Elizabeth; BMJ Publishing; Brigham and Women's Hospital, Department of Neurology<br>Lundh, Andreas; University of Southern Denmark, Department of Clinical Research; Copenhagen University Hospital, Department of Respiratory

Medicine and Infectious Diseases
Meyerowitz-Katz, Gideon; University of Wollongong, School of Health and Society
Mol, Ben; Monash Medical School, OB/GYN
O'Connell, Neil; Brunel University, Department of Clinical Science
Parker, Lisa; The University of Sydney, School of Pharmacy
Redman, Barbara; New York University Grossman School of Medicine, Division of Medical Ethics
Seidler, Lene ; University of Sydney, NHMRC Clinical Trials Centre
Sheldrick, Kyle; University of New South Wales, Faculty of Medicine
Sydenham, Emma; Cochrane, Cochrane Central Production Service
Torgerson, David; The University of York, York Trials Unit
van Wely, Madelon; Amsterdam University Medical Centres, Netherlands Satellite of the Cochrane Gynaecology and Fertility Group
Wang, Rui; Monash University, Department of Obstetrics and Gynaecology
Bero, Lisa; University of Colorado - Anschutz Medical Campus, Center for Bioethics and Humanities
Kirkham, Jamie J.; Manchester University, Biostatistics

Keywords: Randomized Controlled Trial, Systematic Review, STATISTICS & RESEARCH METHODS

SCHOLARONE™ Manuscripts

Protocol for the development of a tool (INSPECT-SR) to identify problematic randomised controlled trials in systematic reviews of health interventions.

Jack Wilkinson[1†], Calvin Heal[1], George A. Antoniou[2,3], Ella Flemyng[4], Zarko Alfirevic[5], Alison Avenell[6], Virginia Barbour[7], Nicholas J L Brown[8], John Carlisle[9], Mike Clarke[10], Patrick Dicker[11], Jo Dumville[12,13], Andrew Grey[14], Steph Grohmann[4], Lyle C Gurrin[15], Jill A Hayden[16], James Heathers[17], Kylie E Hunter[18], Toby Lasserson[4], Emily Lam[19], Sarah Lensen[20], Tianjing Li[21], Wentao Li[22], Elizabeth Loder[23, 24], Andreas Lundh[25, 26], Gideon Meyerowitz-Katz[27], Ben W Mol[28,29], Neil E O' Connell[30], Lisa Parker[31], Barbara K. Redman[32], Anna Lene Seidler[18], Kyle A Sheldrick[33], Emma Sydenham[34], David J Torgerson[35], Madelon van Wely[36, 37], Rui Wang[22], Lisa Bero[38]*, Jamie J Kirkham[1]*

*Joint senior authorship

† Corresponding author: jack.wilkinson@manchester.ac.uk

[1] Centre for Biostatistics, The University of Manchester, Manchester Academic Health Science Centre, Manchester, UK.

[2] Manchester Vascular Centre, Manchester University NHS Foundation Trust, Manchester, UK.

[3] Division of Cardiovascular Sciences, School of Medical Sciences, Manchester Academic Health Science Centre, The University of Manchester, Manchester, UK.

[4] Evidence Production and Methods Directorate, Cochrane Central Executive, London, UK.

[5] Emeritus Professor, University of Liverpool, UK.

[6] Health Services Research Unit, University of Aberdeen, UK.

[7] Medical Journal of Australia, Sydney, Australia.

[8] Department of Psychology, Linnaeus University, Växjö, Sweden.

[9] Perioperative Medicine, Torbay hospital, UK.

[10] Northern Ireland Methodology Hub, Queen's University Belfast, Northern Ireland.

[11] Department of Epidemiology and Public Health, Royal College of Surgeons in Ireland.

For peer review only

[12] Division of Nursing, Midwifery & Social Work, School of Health Sciences, The University of Manchester, Manchester, UK.

[13] NIHR Manchester Biomedical Research Centre, Manchester University NHS Foundation Trust, Manchester Academic Health Science Centre, Manchester, UK.

[14] Department of Medicine, University of Auckland, Auckland, New Zealand.

[15] School of Population and Global Health, The University of Melbourne, Australia

[16] Department of Community Health & Epidemiology, Faculty of Medicine, Dalhousie University, Canada.

[17] SafeBeat Rx Inc, USA.

[18] Evidence Integration, NHMRC Clinical Trials Centre, University of Sydney, Sydney, Australia.

[19] Independent lay member, unaffiliated, Cheshire, UK.

[20] Department of Obstetrics and Gynaecology, Royal Women's Hospital, University of Melbourne, Melbourne, Australia

[21] Department of Ophthalmology, University of Colorado Anschutz Medical Campus.

[22] Department of Obstetrics and Gynaecology, Monash University, VIC, Australia.

[23] The BMJ, London, UK.

[24] Department of Neurology, Brigham and Women's Hospital, Boston, MA, USA.

[25] Cochrane Denmark & Centre for Evidence-Based Medicine Odense, Department of Clinical Research, University of Southern Denmark, Denmark.

[26] Department of Respiratory Medicine and Infectious Diseases, Copenhagen University Hospital, Bispebjerg and Frederiksberg, Denmark.

[27] School of Health and Society, University of Wollongong, Wollongong, Australia.

[28] Monash University, Clayton, Victoria , Australia.

[29] Aberdeen Centre for Women's Health Research, University of Aberdeen, Aberdeen, UK.

[30] Centre for Health and Wellbeing across the Lifecourse, Dept of Health Sciences, Brunel University London, UK.

[31] School of Pharmacy, Charles Perkins Centre, The University of Sydney, Australia.

[32] Division of Medical Ethics, New York University Grossman School of Medicine, New York, NY.

For peer review only

[33] Faculty of Medicine, University of New South Wales, Australia.

[34] Cochrane Central Production Service, UK.

[35] York Trials Unit, Dept of Health Sciences, University of York, UK.

[36] Cochrane Gynaecology and Fertility Satellite and Cochrane Sexually Transmitted Infection Group, Amsterdam.

[37] Reproduction and Development Research Institute, Amsterdam University Medical Center, The Netherlands.

[38] University of Colorado Anschutz Medical Campus, Colorado, USA.

Word count: Main text 3875 words

For peer review only

Abstract

Introduction

Randomised controlled trials (RCTs) inform healthcare decisions. It is now apparent that some published RCTs contain false data and some appear to have been entirely fabricated. Systematic reviews are performed to identify and synthesise all RCTs that have been conducted on a given topic. While it is usual to assess methodological features of the RCTs in the process of undertaking a systematic review, it is not usual to consider whether the RCTs contain false data. Studies containing false data therefore go unnoticed and contribute to systematic review conclusions. The INSPECT-SR project will develop a tool to assess the trustworthiness of RCTs in systematic reviews of healthcare related interventions.

Methods and analysis

The INSPECT-SR tool will be developed using expert consensus in combination with empirical evidence, over five stages: 1) a survey of experts to assemble a comprehensive list of checks for detecting problematic RCTs, 2) an evaluation of the feasibility and impact of applying the checks to systematic reviews, 3) a Delphi survey to determine which of the checks are supported by expert consensus, culminating in 4) a consensus meeting to select checks to be included in a draft tool and to determine its format, 5) prospective testing of the draft tool in the production of new health systematic reviews, to allow refinement based on user feedback. We anticipate that

the INSPECT-SR tool will help researchers to identify problematic studies, and will help patients by protecting them from the influence of false data on their healthcare.

Keywords: randomised controlled trials, research integrity, data fabrication, data falsification, research misconduct

Background

Randomised controlled trials (RCTs) are used to assess the benefits and harms of interventions. Systematic reviews of health and care interventions include all RCTs relating to the review question, synthesising the evidence to arrive at an overall conclusion about whether an intervention is effective works and whether it causes harm. It is well-recognised that some RCTs included in systematic reviews of health interventions are unreliable due to methodological limitations. However, relatively little attention has been given to the fact that some published RCTs are untrustworthy not because of methodological limitations, but rather because they contain false data, and may not have taken place at all. This could be due to research misconduct (including fabrication or falsification of data) or critical errors which would not be identified during established assessments of methodological quality.

A recent illustrative example is ivermectin for treatment and prophylaxis of COVID-19. Several systematic reviews evaluating ivermectin for COVID-19 concluded that the drug reduced mortality (1, 2). Subsequently, it became apparent that these systematic reviews had accidentally included RCTs which appear to have been partially or wholly fabricated(3). For example, the spreadsheet purportedly containing the data from one of these trials featured repeating blocks of data(4). Once these RCTs were excluded, the conclusion of a clear benefit of ivermectin was no longer supported(5). The threat posed by RCTs of questionable veracity is not confined to a particular field of medicine or health. For example, studies of this nature have been identified in systematic reviews of Vitamin K for prevention of fractures(6, 7), tranexamic acid for prevention of postpartum haemorrhage(8), and psychological therapies for management of chronic pain(9).

While RCTs are routinely appraised on the basis of both their internal and external validity during the systematic review process, this appraisal is predicated on the assumption that the studies are genuine; the veracity of the studies is not formally assessed. It is now clear that many studies of questionable veracity describe sound methodology, and so are not flagged by critical appraisal frameworks such as Risk of Bias tools. This prompts the question of how we should assess the veracity of RCTs during the systematic review process. The overall aim of the INSPECT-SR (INveStigating ProblEmatic Clinical Trials in Systematic Reviews) project is to develop and evaluate a tool for identifying these *problematic studies* in the context of systematic reviews of RCTs of health interventions. In the following, we give an overview of the project methods.

For peer review only - http://bmjopen.bmj.com/site/about/guidelines.xhtml

Methods/design

The INSPECT-SR tool will be developed using expert consensus in combination with empirical evidence, over five stages (Figure 1): 1) a survey of experts to assemble a comprehensive list of checks for detecting problematic RCTs, 2) an evaluation of the feasibility and impact of applying the checks to RCTs in systematic reviews, 3) a Delphi survey to determine which of the checks are supported by expert consensus, culminating in 4) a consensus meeting to select checks to be included in a draft tool and to determine its format, and finally 5) prospective testing of the draft tool in the production of new health systematic reviews, to allow refinement based on user feedback.

Working definition of a 'problematic study'

The Cochrane policy on Managing Potentially Problematic Studies (10, 11) defines a problematic study as "any published or unpublished study where there are serious questions about the trustworthiness of the data or findings, regardless of whether the study has been formally retracted." We adopt this as a working definition at the outset of the INSPECT-SR project, noting that the project involves the identification of criteria for evaluating 'trustworthiness'. Criteria under consideration could include statistical checks of data and results, aspects of research governance such as ethical approval, presence of plagiarised content, plausibility of the study conduct, or the track record of the research team. Criteria relating to internal or external validity of results produced by RCTs, such as those included in Risk of Bias(12), Risk of Bias 2(13), or GRADE(14) frameworks, are not within the scope of the INSPECT-SR project. The INSPECT-SR tool will be designed to be used alongside these established critical appraisal frameworks. Assessment of conflicts of interest will be covered by TACIT (Tool for Addressing Conflicts of Interest in Trials)(15) and will not be covered by INSPECT-SR.

INSPECT-SR working group

The INSPECT-SR working group comprises a steering group, an expert advisory panel, a Delphi panel, and additional collaborators. The steering group includes experts in research integrity, clinical trials methodology, systematic reviews, consensus methodology, and methodological guideline development. They will coordinate the development and evaluation of INSPECT-SR. A larger expert advisory panel has been established to provide advice and to contribute throughout the project. This expert panel has been selected to represent a diverse range of relevant expertise and experience. This includes methodologists, research integrity specialists, public contributors, researchers with experience of investigating potentially problematic studies, experts in systematic reviews, and journal editors. Members of the steering group and expert advisory panel were involved as participants in the Stage 1 survey, and may be eligible to participate in the Stage 3 Delphi survey and consensus meeting. Additional collaborators are involved in Stage 2, and may be eligible to participate in the Stage 3 Delphi survey.

For peer review only - http://bmjopen.bmj.com/site/about/guidelines.xhtml

Stage 1: Survey of experts to assemble an extensive list of checks

Overall design

The Stage 1 survey of experts has been completed at the time of writing, and a short protocol for the survey has been posted online (https://osf.io/6pmx5/). We describe the methods briefly here. The aim of Stage 1 was to create an extensive list of checks for identifying problematic research studies, which could be taken forward for evaluation in stages 2 and 3. In Stage 1, we did not restrict our focus to checks applicable to or designed for RCTs specifically. Instead, we sought to identify checks applicable to any research design, so that these could be subsequently evaluated for their applicability to RCTs.

We assembled an initial list of 102 checks that could be used to assess potentially problematic studies. The initial list included checks identified in a recent scoping review(16), a recent qualitative study of experts(17) and additional methods known to the research team (for example, JW undertakes integrity investigations for scientific journals and publishers, and added checks known to him as a result of this work). The list was grouped into several preliminary domains, as shown in Table 1 (adapted from https://osf.io/6pmx5/).

We incorporated the list in an online survey in Qualtrics (available at https://osf.io/6pmx5/) to identify checks which had not already been included on the list, and to allow respondents to comment on the checks which were on the list.

The survey asked participants about their experience in assessing potentially problematic studies and to state the country in which they primarily work, before presenting them with the initial list of 102 checks. Each item was presented alongside a free-text box, and participants were advised to comment on any aspect if they wished to do so. At the end of the list, participants were asked whether they were aware of any other checks that had not featured on the list, and were presented with a free text box to describe these.

For peer review only

Table 1: Frequency of items grouped into preliminary domains included in an online survey of experts (Stage 1).

**Preliminary domain**	**Frequency of items per domain**
1. Inspecting results in the paper	26
2. Inspecting the research team	17
3. Inspecting conduct, governance and transparency	19
4. Inspecting text and publication details	7
5. Inspecting individual participant data	33
	102

For peer review only

Participants and recruitment

People with expertise or experience in assessing potentially problematic studies, before or after publication, were eligible to participate in the survey. We identified eligible individuals primarily through professional networks, including promotion of the project via conference presentations, and by social media. Members of the steering group and expert advisory panel were invited to participate. We invited eligible individuals by personalised email, and asked whether they could suggest any other potential participants. We attempted to achieve global representation by monitoring the countries in which the respondents worked as responses accumulated, and renewing our efforts to identify and recruit respondents from underrepresented regions. We targeted a minimum sample size of 50, but obtained as many responses as possible.

Analysis and next steps

Descriptive analysis of the survey participants (country of work, experience with assessing potentially problematic studies) and responses will be performed. Additional items suggested by respondents, and comments made on existing items, will be summarised. Based on the survey responses further items will be added to the list, and the wording of existing items will be amended, subject to review by the steering group and expert advisory panel members. The updated list will be taken forward to Stages 2 and 3.

Checks categorised in Domain 5 (Inspecting individual participant data, see Table 1) may only be performed when the underlying dataset for an RCT can be obtained. An extension to the INSPECT-SR tool containing Domain 5 checks is in development (working name *INSPECT-IPD*). The development of INSPECT-IPD requires a different approach to the main INSPECT-SR tool (application of checks to a large sample of individual participant datasets, and a distinct Delphi panel). The remainder of this protocol describes the development of the INSPECT-SR tool, which will include checks in Domains 1 to 4 only.

<u>Stage 2: Retrospective application of the list of items to systematic reviews</u>

Overall design

We will apply the full list of checks we have identified to RCTs in a large sample of systematic reviews of interventions published in the Cochrane library, in order to evaluate their feasibility and impact.

Review selection

We will use a sample of 50 Cochrane Reviews. This sample size has been selected on a pragmatic basis, to allow a sufficient number of applications of the checks to evaluate feasibility and to characterise the impact on results, while remaining achievable. Stage 2 will be undertaken

For peer review only

as a large collaborative enterprise, with steering group members, expert advisory panel members, and additional collaborators who have expressed an interest in participating, each applying the full list of checks to the RCTs in a small number of Cochrane Reviews.

We will endeavour to match assessors to topic areas with which they have familiarity, as this reflects how the final INSPECT-SR tool would be used. We will ask each assessor to state a broad topic area relating to their expertise. We will then identify the most recent Cochrane Review relating to this topic and meeting the eligibility requirements. Where an assessor does not have a particular topic of interest, we will select a topic in order to achieve broad coverage of subjects, and will identify the most recently published Cochrane Review meeting the eligibility requirements. To be eligible, a Review cannot be authored or co-authored by the assessor, out of concern that this could introduce an incentive to overlook problematic features of included studies. Similarly, the Review should not contain RCTs authored or coauthored by the assessor. The Review must also contain at least one meta-analysis containing between one and five RCTs as a feasibility constraint. We also require that the Review has not undergone an assessment to identify potentially problematic studies already, as this may have resulted in removal of problematic trials from the meta-analyses. We acknowledge however that this final criterion may frequently be unclear.

Data capture

A bespoke data capture form has been produced. Assessors will extract data for each RCT contained in the first meta-analysis in the Cochrane Review which includes between 1 and 5 RCTs. Assessors will initially record their level of familiarity with the topic of the Review (little or no familiarity, some familiarity, high familiarity), and basic information about each RCT, including a study ID based on the names of the first authors of the Review and of the trial, and years of publication of both, and the year of publication of the RCT. Assessors will then extract data for that RCT from the meta-analysis, including sample size per treatment arm and outcome data per treatment arm (e.g. mean and standard deviation for each treatment arm for continuous outcomes, and frequency of events for binary outcomes). The Risk of Bias assessments for that RCT from the Review will be extracted for each domain, as will the corresponding GRADE assessment for the meta-analysis (if there is one).

Assessors will then attempt to apply items from the list of checks from Stage 1 to the RCT. Assessors will be given the opportunity to apply each check, with the exception of checks which require authors of the RCT to be contacted. For each check, assessors will select a response from the options “not feasible”, “passed”, “possibly fail” or “fail”. A free text box will be available for each check so that the assessor may record the reason for their assessment. Finally, having worked through the list of checks, the assessor will record whether they have concerns about the authenticity of the RCT (with options “no”, “some concerns”, “serious concerns”, or “don’t know”), whether they performed any additional checks not included in the list (and if so, what these checks were and what the outcomes were), as well as being given the opportunity to make any additional comments and to estimate how long it took to perform the assessment.

For peer review only

To assist with applying the checks, each assessor will be provided with a guidance document briefly explaining the rationale for each check and instructions on how to apply them. An Excel workbook will be supplied, which can be used to perform some of the statistical checks.

Statistical analysis

We will calculate the frequency of each response option for each check (how often each was considered infeasible, how often each one was failed, possibly failed or passed). We will summarise the overall RCT-level assessments of the assessors after applying the checks (whether or not they had concerns about authenticity). We will evaluate the impact of removing trials flagged by each item, by comparing the data included in the primary meta-analysis before and after the application of the method (e.g. numbers of trials, numbers of events, sample size) as well as the results (changes in pooled estimate, confidence interval width, heterogeneity). We will visualise the clustering of checks, by plotting trial-level assessments for each check in an array. We will consider the relationship between the assessments and the risk of bias (for each domain) in the reviews, to understand the relationship between indicators of problems on the one hand and assessments of evidence quality on the other. This will be undertaken using multinomial regression to assess the association between assessment and risk of bias ratings for each risk of bias domain. GRADE assessments refer to collections of trials rather than individual trials, and so we will use ordinal regression to assess the association between the number of trials in the meta-analysis flagged and the GRADE rating.

For peer review only

Stage 3: Delphi survey

Overall design

A two-round Delphi survey will be conducted to determine which checks are supported by expert consensus.

Participants and recruitment

Delphi participants will be identified through professional networks of the steering group and expert advisory panel. We will also invite eligible individuals identified and involved in previous stages of the project. We will recruit individuals representing key stakeholder groups, including: individuals with experience or expertise in assessing problematic studies, journal editors, research integrity specialists, systematic reviewers, clinical trialists, and methodologists. We will

categorise participants into two larger groups: 1) individuals with expertise or experience in assessing potentially problematic studies and 2) potential users of a tool for assessing potentially problematic studies, noting that participants may be included in both categories. Individuals will be invited via personalised email describing the Delphi survey in the context of the wider INSPECT-SR development project. We will monitor recruitment across stakeholder groups and geographical location, and will attempt to improve recruitment for groups in which recruitment numbers are low by targeting potential participants in these groups. We consider at least 30 expert participants in each of the two participant groups (experts and potential users) to represent the minimum for a credible Delphi. However, ideally we will aim for a minimum of 100 participants overall.

Selection of items

The list of items obtained from Stage 1 will be entered into the Delphi survey. Checks will be categorised and presented in several domains (see Table 1 for the preliminary categorisation scheme, used in Stage 1 but subject to change as the project evolves). We will develop suitable language to clearly describe the checks. The list will be approved by the expert advisory panel before we launch the Delphi survey, including review by public contributors to confirm clarity. We will write an explanation to accompany each item, which participants may review if they are unsure of its meaning.

Round 1

We will send participants a personalised email outlining the project, together with a link to the survey, which will be implemented online using suitable software. The survey will include the list of checks. In Round 1, respondents will be asked for basic demographic information, to allow categorisation based on domain(s) of expertise. Respondents will be asked to score each check 1 (lowest score) to 9 (highest score) in two dimensions: usefulness and feasibility. Usefulness will relate to the potential effectiveness of the check for detecting a problematic study. Feasibility will relate to the perceived ease of implementation of each check. Participants will also be given the option to indicate that they do not know whether a check is useful or feasible (because, for example, they are unfamiliar with the approach or lack expertise to comment on a particular check). A free-text box will be provided with each check, so that participants may leave any general comments (such as an explanation for their assessment, or suggestions to modify the wording). Round 1 participants will be invited to suggest additional checks.

Round 2

For peer review only

In Round 2, we will add any suggested additional checks to the list (subject to review by the steering group and expert advisory panel), and for each item respondents will be presented with both their own scores (1 to 9) and the distribution of scores from the previous round. Participants who were invited to participate in Round 1 but who did not respond will be invited to the Round 2 survey, and will be presented with the distribution of scores from the previous round only. Participants will be asked to provide a new score in light of this information. The Round 2 survey will include a free-text box for each check so that participants may elaborate on their responses.

Analysis

Check-specific scores from Round 2 will be summarised for the overall Delphi panel and by stakeholder group. Any items that meet a consensus criterion, defined as scoring 7 or more by at least 80% of participants overall or in one or more stakeholder groups for usefulness, will be automatically considered during the Stage 4 consensus meeting. Items failing to meet a consensus criterion will be discussed by the steering group and expert advisory panel in light of the Stage 2 application exercise, and will be considered for inclusion in the Stage 4 consensus meetings. Feasibility scores will be summarised for each check, and will be used in Stage 4.

For peer review only

Stage 4: Consensus meetings

Consensus meetings will be held to finalise the checks to be included in the draft INSPECT-SR tool. We anticipate that multiple meetings will be necessary in order to accommodate international time differences. Meetings may be virtual, in-person, or a combination of both. At these meetings, the results of the Stage 2 application exercise and Stage 3 Delphi survey will be discussed, with the purpose of finalising the items to be included in the draft INSPECT-SR tool. The feasibility assessments from the Stage 2 application exercise and Stage 3 Delphi survey will be considered for all items discussed. Items that are considered useful but challenging to implement may not be incorporated into the main tool, but instead included as an optional or recommended check in the accompanying guidance document. Participants will be invited to reflect the range of key stakeholder groups, as described above. We anticipate that 20 to 30 participants will participate in the consensus meetings, with ten to fifteen participants representing each of the two main participant groups (experts and potential users). In addition to determining the checks to include in the tool, it will be necessary to determine its form and structure, and the recommended process for applying it during the systematic review process. It may be necessary to hold additional meetings focussed on these questions.

Stage 5: Prospective testing of draft tool

Overall design

In collaboration with systematic reviewers, we will prospectively evaluate the draft tool by using it in the production of a cohort of new systematic reviews and systematic review updates. The impact of the draft tool's Impact on Review conclusions will be assessed in the same way as in Stage 2. We will assess feasibility and usability by implementing surveys regarding experiences of use. Separate surveys will be designed for review authors and, for Cochrane Reviews, editors. These will explore ease of implementation, barriers to use, and suggestions for improvement. In addition to user-level data, we will capture data relating to the individual reviews in which the tool was implemented, as each one represents a potentially informative case study. We will undertake additional qualitative interviews with users during this testing phase, to capture additional feedback.

We will aim to include a variety of topic areas in this testing phase. Stage 5 will culminate in a user workshop, including review editors and review authors involved in testing the tool.

User workshop

Findings from the surveys will be fed back to participants as part of a user workshop. The workshop might be virtual, in-person, or a combination of both. Participants will share their experiences of using the tool, and make recommendations for refinement. The discursive format of the meeting is intended to reveal additional information about the experience of users that could not be easily captured via the surveys. We will invite both authors and editors involved in the testing phase to participate. The findings of the testing phase will be used to make final modifications to the tool for usability. We will use the results to produce guidance relating to use of the tool in practice. Alongside Stage 5, as we gather user data, we will produce training materials (to be delivered as workshops and as an online training module) to familiarise systematic review authors and editors with the tool. These will be finalised in light of the findings from the user workshop.

Patient and public involvement

An outline for the project was reviewed and commented by patient partners prior to grant submission. The expert advisory panel includes two lay members, who are given equal opportunity to contribute to the design and dissemination of all work packages. One lay member is acting as a co-author on the current manuscript.

Conclusion

For peer review only

Systematic reviews of health interventions are considered to represent a very high standard of evidence and frequently inform policy and practice. However, because the veracity of included RCTs is not usually considered, systematic reviews may unintentionally act as a pipeline for false data with the risk that this will influence care. While the need to prevent problematic studies from contributing to systematic reviews is recognised, with several recent laudable efforts to tackle the issue (18-20), there is currently limited agreement on how this should be done. The INSPECT-SR project will develop a tool for evaluating the trustworthiness studies, backed by empirical evidence and expert consensus. We anticipate the draft tool will be available early 2024, and the final tool will be available late 2024.

Funding

This research is funded by the NIHR Research for Patient Benefit programme (NIHR203568). The views expressed are those of the author(s) and not necessarily those of the NIHR or the Department of Health and Social Care.

Declaration of interests

JW, CH, GA, LB, JJK declare funding from NIHR (NIHR203568) in relation to this work. LB additionally declares The University of Colorado receives remuneration for service as Senior Research Integrity Editor, Cochrane. WL, ALS, and RW declare funding from Australian National Health and Medical Research Council Investigator Grants (GNT2016729, GNT2009432, GNT2009767). EF, SG, TLa declare employment by Cochrane. Tla additionally declares authorship of a chapter in the Cochrane Handbook for Systematic Reviews of Interventions and that he is a developer of standards for Cochrane intervention reviews (MECIR). ES declares that she was a member of the Cochrane scientific misconduct policy advisory group. ZA declares he is a member of the Cochrane Library Editorial Board, and PI on a grant from Children Investment Foundation Fund to University of Liverpool to investigate research integrity of clinical trials related to nutritional supplements in pregnancy. TLi is supported by grant UG1 EY020522 from the National Eye Institute, National Institutes of Health. MC declares that he is Co-ordinating Editor for the Cochrane Methodology Review Group. AA declares that The Health Services Research Unit, University of Aberdeen, is funded by the Health and Social Care Directorates of the Scottish Government.

Ethical approval

For peer review only

The University of Manchester ethics decision tool was used, and this returned the result that ethical approval was not required for this project (30th September 2022), which incorporates secondary research and surveys of professionals about subjects relating to their expertise.

References

1. Bryant A, Lawrie TA, Fordham EJ. Ivermectin for Prevention and Treatment of COVID-19 Infection: A Systematic Review, Meta-analysis, and Trial Sequential Analysis to Inform Clinical Guidelines. American Journal of Therapeutics, 28, e434-e460, July 2021. Am J Ther. 2021;28(5):e573-e6.
2. Hill A, Garratt A, Levi J, Falconer J, Ellis L, McCann K, et al. Meta-analysis of Randomized Trials of Ivermectin to Treat SARS-CoV-2 Infection. Open Forum Infect Dis. 2021;8(11):ofab358.
3. Lawrence JM, Meyerowitz-Katz G, Heathers JAJ, Brown NJL, Sheldrick KA. The lesson of ivermectin: meta-analyses based on summary data alone are inherently unreliable. Nat Med. 2021;27(11):1853-4.
4. Brown NJL. http://steamtraen.blogspot.com/2021. [cited 2023]. Available from: http://steamtraen.blogspot.com/2021/07/Some-problems-with-the-data-from-a-Covid-study.html.
5. Hill A, Mirchandani M, Pilkington V. Ivermectin for COVID-19: Addressing Potential Bias and Medical Fraud. Open Forum Infect Dis. 2022;9(2):ofab645.
6. Cockayne S, Adamson J, Lanham-New S, Shearer MJ, Gilbody S, Torgerson DJ. Vitamin K and the prevention of fractures: systematic review and meta-analysis of randomized controlled trials. Arch Intern Med. 2006;166(12):1256-61.
7. Grey A, Avenell A, Bolland M. Revised Meta-analysis of Vitamin K and Fractures. JAMA Intern Med. 2018;178(8):1135.
8. Ker K, Shakur H, Roberts I. Does tranexamic acid prevent postpartum haemorrhage? A systematic review of randomised controlled trials. BJOG. 2016;123(11):1745-52.
9. Williams ACC, Fisher E, Hearn L, Eccleston C. Psychological therapies for the management of chronic pain (excluding headache) in adults. Cochrane Database Syst Rev. 2020;8(8):CD007407.
10. Cochrane. Cochrane Database of Systematic Reviews: editorial policies Cochrane Library [Available from: https://www.cochranelibrary.com/cdsr/editorial-policies.
11. Boughton SL, Wilkinson J, Bero L. When beauty is but skin deep: dealing with problematic studies in systematic reviews. Cochrane Database Syst Rev. 2021;6(6):ED000152.
12. Higgins JP, Altman DG, Gotzsche PC, Juni P, Moher D, Oxman AD, et al. The Cochrane Collaboration's tool for assessing risk of bias in randomised trials. BMJ. 2011;343:d5928.
13. Sterne JAC, Savovic J, Page MJ, Elbers RG, Blencowe NS, Boutron I, et al. RoB 2: a revised tool for assessing risk of bias in randomised trials. BMJ. 2019;366:l4898.
14. Guyatt GH, Oxman AD, Kunz R, Vist GE, Falck-Ytter Y, Schünemann HJ. What is “quality of evidence” and why is it important to clinicians? BMJ. 2008;336(7651):995-8.
15. Tool for Addressing Conflicts of Interest in Trials [Available from: https://tacit.one/.
16. Bordewijk EM, Li W, van Eekelen R, Wang R, Showell M, Mol BW, et al. Methods to assess research misconduct in health-related research: A scoping review. J Clin Epidemiol. 2021;136:189-202.
17. Parker L, Boughton S, Lawrence R, Bero L. Experts identified warning signs of fraudulent research: a qualitative study to inform a screening tool. J Clin Epidemiol. 2022;151:1-
18. Mol BW, Lai S, Rahim A, Bordewijk EM, Wang R, van Eekelen R, et al. Checklist to assess Trustworthiness in RAndomised Controlled Trials (TRACT checklist): concept proposal and pilot. Res Integr Peer Rev. 2023;8(1):6.

For peer review only

19. Weibel S, Popp M, Reis S, Skoetz N, Garner P, Sydenham E. Identifying and managing problematic trials: A research integrity assessment tool for randomized controlled trials in evidence synthesis. Res Synth Methods. 2023;14(3):357-69.
20. Grey A, Bolland MJ, Avenell A, Klein AA, Gunsalus CK. Check for publication integrity before misconduct. Nature. 2020;577(7789):167-9.

Figure captions:

Figure 1: INSPECT-SR development process.

For peer review only

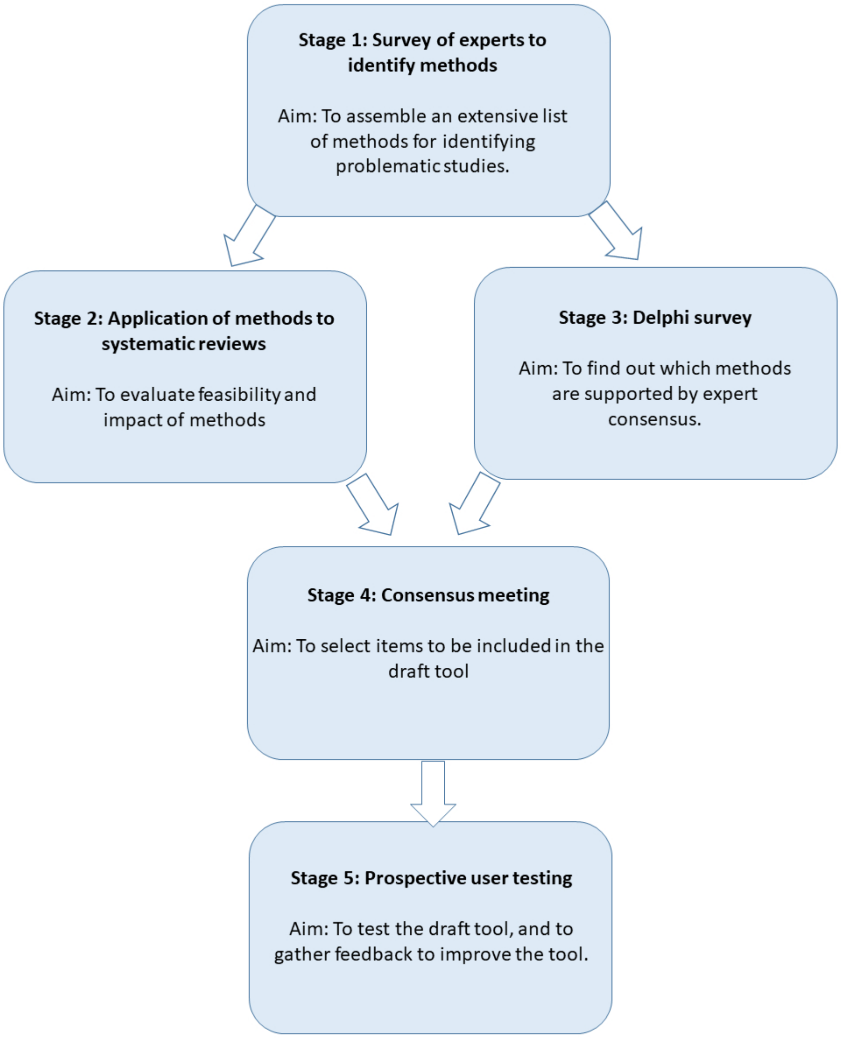

INSPECT-SR development process

302x338mm (96 x 96 DPI)

BMJ Open

Protocol for the development of a tool (INSPECT-SR) to identify problematic randomised controlled trials in systematic reviews of health interventions

Journal:	*BMJ Open*
Manuscript ID	bmjopen-2024-084164.R1
Article Type:	Protocol
Date Submitted by the Author:	29-Jan-2024
Complete List of Authors:	Wilkinson, Jack; The University of Manchester, Centre for Biostatistics, Manchester Academic Health Science Centre<br>Heal, Calvin; The University of Manchester, Centre for Biostatistics, Manchester Academic Health Science Centre; Salford Royal NHS Foundation Trust,<br>Antoniou , George ; Manchester University NHS Foundation Trust, Manchester Vascular Centre; The University of Manchester Faculty of Biology Medicine and Health, Division of Cardiovascular Sciences, School of Medical Sciences, Manchester Academic Health Science Centre<br>Flemyng, Ella; Cochrane, Evidence Production and Methods Directorate<br>Alfirevic, Zarko; University of Liverpool, Department of Women's and Children's Health<br>Avenell, Alison; University of Aberdeen, Health Services Research Unit<br>Barbour, Ginny; Medical Journal of Australia<br>Brown, Nicholas; Linnaeus University, Department of Psychology<br>Carlisle, John; Torbay Hospital, Anaesthesia and Critical Care<br>Clarke, Mike; Queen's University Belfast, Northern Ireland Methodology Hub<br>Dicker, Patrick; Royal College of Surgeons in Ireland, Department of Epidemiology and Public Health<br>Dumville, Jo C.; The University of Manchester, Division of Nursing, Midwifery and Social Work, School of Health Sciences; Manchester University NHS Foundation Trust, Manchester Academic Health Science Centre<br>Grey, Andrew; University of Auckland, Department of Medicine<br>Grohmann, Steph; Cochrane<br>Gurrin, Lyle; University of Melbourne School of Population and Global Health<br>Hayden, Jill; Dalhousie University, Community Health & Epidemiology<br>Heathers, James; SafeBeat Rx Inc<br>Hunter, Kylie; NHMRC Clinical Trials Centre, University of Sydney<br>Lasserson, Toby; Cochrane, Evidence Production and Methods Directorate<br>Lam, Emily; Independent Lay Member<br>Lensen, Sarah; University of Melbourne, Obstetrics and Gynaecology<br>Li, Tianjing; University of Colorado, Department of Ophthalmology<br>Li, Wentao; Monash University, Department of Obstetrics and Gynecology<br>Loder, Elizabeth; BMJ Publishing; Brigham and Women's Hospital,

	Department of Neurology<br>Lundh, Andreas; University of Southern Denmark, Centre of Evidence-Based Medicine Odense and Cochrane Denmark, Department of Clinical Research; Copenhagen University Hospital Bispebjerg and Frederiksberg, Department of Respiratory Medicine and Infectious Diseases<br>Meyerowitz-Katz, Gideon; University of Wollongong, School of Health and Society<br>Mol, Ben; Monash University, Department of Obstetrics and Gynaecology<br>O'Connell, Neil; Brunel University, Department of Clinical Science<br>Parker, Lisa; The University of Sydney, School of Pharmacy<br>Redman, Barbara; New York University Grossman School of Medicine, Division of Medical Ethics<br>Seidler, Lene ; University of Sydney, NHMRC Clinical Trials Centre<br>Sheldrick, Kyle; University of New South Wales, Faculty of Medicine<br>Sydenham, Emma; Cochrane, Cochrane Central Production Service<br>Torgerson, David; The University of York, York Trials Unit<br>van Wely, Madelon; Amsterdam University Medical Centres, Netherlands Satellite of the Cochrane Gynaecology and Fertility Group<br>Wang, Rui; Monash University, Department of Obstetrics and Gynaecology<br>Bero, Lisa; University of Colorado - Anschutz Medical Campus, Center for Bioethics and Humanities<br>Kirkham, Jamie J.; The University of Manchester, Centre for Biostatistics, Manchester Academic Health Science Centre

**Primary Subject Heading**:	Research methods
Secondary Subject Heading:	Medical publishing and peer review
Keywords:	Randomized Controlled Trial, Systematic Review, STATISTICS & RESEARCH METHODS

SCHOLARONE™
Manuscripts

Protocol for the development of a tool (INSPECT-SR) to identify problematic randomised controlled trials in systematic reviews of health interventions

Jack Wilkinson[1†], Calvin Heal[1], George A. Antoniou[2,3], Ella Flemyng[4], Zarko Alfirevic[5], Alison Avenell[6], Virginia Barbour[7], Nicholas J L Brown[8], John Carlisle[9], Mike Clarke[10], Patrick Dicker[11], Jo Dumville[12,13], Andrew Grey[14], Steph Grohmann[4], Lyle C Gurrin[15], Jill A Hayden[16], James Heathers[17], Kylie E Hunter[18], Toby Lasserson[4], Emily Lam[19], Sarah Lensen[20], Tianjing Li[21], Wentao Li[22], Elizabeth Loder[23, 24], Andreas Lundh[25, 26], Gideon Meyerowitz-Katz[27], Ben W Mol[28,29], Neil E O' Connell[30], Lisa Parker[31], Barbara K. Redman[32], Anna Lene Seidler[18], Kyle A Sheldrick[33], Emma Sydenham[34], David J Torgerson[35], Madelon van Wely[36, 37], Rui Wang[22], Lisa Bero[38]*, Jamie J Kirkham[1]*

[1] Centre for Biostatistics, The University of Manchester, Manchester Academic Health Science Centre, Manchester, UK.

[2] Manchester Vascular Centre, Manchester University NHS Foundation Trust, Manchester, UK.

[3] Division of Cardiovascular Sciences, School of Medical Sciences, Manchester Academic Health Science Centre, The University of Manchester, Manchester, UK.

[4] Evidence Production and Methods Directorate, Cochrane Central Executive, London, UK.

[5] Emeritus Professor, University of Liverpool, Liverpool, UK.

[6] Health Services Research Unit, University of Aberdeen, Aberdeen, UK.

[7] Medical Journal of Australia, Sydney, Australia.

[8] Department of Psychology, Linnaeus University, Växjö, Sweden.

[9] Perioperative Medicine, Torbay Hospital, UK.

[10] Northern Ireland Methodology Hub, Queen's University Belfast, Northern Ireland.

[11] Department of Epidemiology and Public Health, Royal College of Surgeons in Ireland, Ireland

[12] Division of Nursing, Midwifery & Social Work, School of Health Sciences, The University of Manchester, Manchester, UK.

[13] NIHR Manchester Biomedical Research Centre, Manchester University NHS Foundation Trust, Manchester Academic Health Science Centre, Manchester, UK.

[14] Department of Medicine, University of Auckland, Auckland, New Zealand.

For peer review only

[15] School of Population and Global Health, The University of Melbourne, Australia

[16] Department of Community Health & Epidemiology, Faculty of Medicine, Dalhousie University, Canada.

[17] SafeBeat Rx Inc, USA.

[18] Evidence Integration, NHMRC Clinical Trials Centre, University of Sydney, Sydney, Australia.

[19] Independent lay member, unaffiliated, Cheshire, UK.

[20] Department of Obstetrics and Gynaecology, Royal Women's Hospital, University of Melbourne, Melbourne, Australia

[21] Department of Ophthalmology, University of Colorado Anschutz Medical Campus.

[22] Department of Obstetrics and Gynaecology, Monash University, VIC, Australia.

[23] The BMJ, London, UK.

[24] Department of Neurology, Brigham and Women's Hospital, Boston, MA, USA.

[25] Cochrane Denmark & Centre for Evidence-Based Medicine Odense, Department of Clinical Research, University of Southern Denmark, Denmark.

[26] Department of Respiratory Medicine and Infectious Diseases, Copenhagen University Hospital Bispebjerg and Frederiksberg, Denmark.

[27] School of Health and Society, University of Wollongong, Wollongong, Australia.

[28] Department of Obstetrics and Gynaecology, Monash University, Clayton, Victoria, Australia.

[29] Aberdeen Centre for Women's Health Research, University of Aberdeen, Aberdeen, UK.

[30] Centre for Health and Wellbeing across the Lifecourse, Dept of Health Sciences, Brunel University London, UK.

[31] School of Pharmacy, Charles Perkins Centre, The University of Sydney, Australia.

[32] Division of Medical Ethics, New York University Grossman School of Medicine, New York, NY.

[33] Faculty of Medicine, University of New South Wales, Australia.

[34] Cochrane Central Production Service, UK.

[35] York Trials Unit, Dept of Health Sciences, University of York, UK.

For peer review only

[36] Cochrane Gynaecology and Fertility Satellite and Cochrane Sexually Transmitted Infection Group, Amsterdam.

[37] Reproduction and Development Research Institute, Amsterdam University Medical Center, Netherlands.

[38] University of Colorado Anschutz Medical Campus, Colorado, USA.

*Joint senior authorship

† Corresponding author:

Jack Wilkinson

jack.wilkinson@manchester.ac.uk

For peer review only

Word count (main text): 3875 words

Keywords: randomised controlled trials, research integrity, data fabrication, data falsification, research misconduct

Abstract

Introduction

Randomised controlled trials (RCTs) inform healthcare decisions. It is now apparent that some published RCTs contain false data and some appear to have been entirely fabricated. Systematic reviews are performed to identify and synthesise all RCTs that have been conducted on a given topic. While it is usual to assess methodological features of the RCTs in the process of undertaking a systematic review, it is not usual to consider whether the RCTs contain false data. Studies containing false data therefore go unnoticed and contribute to systematic review conclusions. The INSPECT-SR project will develop a tool to assess the trustworthiness of RCTs in systematic reviews of healthcare related interventions.

Methods and analysis

The INSPECT-SR tool will be developed using expert consensus in combination with empirical evidence, over five stages: 1) a survey of experts to assemble a comprehensive list of checks for detecting problematic RCTs, 2) an evaluation of the feasibility and impact of applying the checks to systematic reviews, 3) a Delphi survey to determine which of the checks are supported by expert consensus, culminating in 4) a consensus meeting to select checks to be included in a draft tool and to determine its format, 5) prospective testing of the draft tool in the production of new health systematic reviews, to allow refinement based on user feedback. We anticipate that the INSPECT-SR tool will help researchers to identify problematic studies and will help patients by protecting them from the influence of false data on their healthcare.

Ethics and dissemination

The University of Manchester ethics decision tool was used, and this returned the result that ethical approval was not required for this project (30th September 2022), which incorporates secondary research and surveys of professionals about subjects relating to their expertise. Informed consent will be obtained from all survey participants. All results will be published as open-access articles. The final tool will be made freely available.

Strengths and limitations of this study

- The tool is being developed using empirical evidence and a large-scale international consensus process.
- Key stakeholders will be involved in the development and dissemination of the tool.
- There is no gold-standard test for inauthentic studies, and so the tool will not be a diagnostic test for fraud; rather, it will help the researcher to make a judgement about trustworthiness.

INTRODUCTION

Randomised controlled trials (RCTs) are used to assess the benefits and harms of interventions. Systematic reviews of health and care interventions include all RCTs relating to the review question, synthesising the evidence to arrive at an overall conclusion about whether an intervention is effective works and whether it causes harm. It is well-recognised that some RCTs included in systematic reviews of health interventions are unreliable due to methodological limitations. However, relatively little attention has been given to the fact that some published RCTs are untrustworthy not because of methodological limitations, but rather because they contain false data, and may not have taken place at all. This could be due to research misconduct (including fabrication or falsification of data) or critical errors which would not be identified during established assessments of methodological quality.

For peer review only

A recent illustrative example is ivermectin for treatment and prophylaxis of COVID-19. Several systematic reviews evaluating ivermectin for COVID-19 concluded that the drug reduced mortality (1, 2). Subsequently, it became apparent that these systematic reviews had accidentally included RCTs which appear to have been partially or wholly fabricated(3). For example, the spreadsheet purportedly containing the data from one of these trials featured repeating blocks of data(4). Once these RCTs were excluded, the conclusion of a clear benefit of ivermectin was no longer supported(5). The threat posed by RCTs of questionable veracity is not confined to a particular field of medicine or health. For example, studies of this nature have been identified in systematic reviews of Vitamin K for prevention of fractures(6, 7), tranexamic acid for prevention of postpartum haemorrhage(8), and psychological therapies for management of chronic pain(9).

While RCTs are routinely appraised on the basis of both their internal and external validity during the systematic review process, this appraisal is predicated on the assumption that the studies are genuine; the veracity of the studies is not formally assessed. It is now clear that many studies of questionable veracity describe sound methodology, and so are not flagged by critical appraisal frameworks such as Risk of Bias tools. This prompts the question of how we should assess the veracity of RCTs during the systematic review process. The overall aim of the INSPECT-SR (INveStigating ProblEmatic Clinical Trials in Systematic Reviews) project is to develop and evaluate a tool for identifying these *problematic studies* in the context of systematic reviews of RCTs of health interventions. In the following, we give an overview of the project methods.

METHODS AND ANALYSIS

The INSPECT-SR tool will be developed using expert consensus in combination with empirical evidence, over five stages (Figure 1): 1) a survey of experts to assemble a comprehensive list of checks for detecting problematic RCTs, 2) an evaluation of the feasibility and impact of applying the checks to RCTs in systematic reviews, 3) a Delphi survey to determine which of the checks are supported by expert consensus, culminating in 4) a consensus meeting to select checks to be included in a draft tool and to determine its format, and finally 5) prospective testing of the draft tool in the production of new health systematic reviews, to allow refinement based on user feedback.

Working definition of a 'problematic study'

The Cochrane policy on Managing Potentially Problematic Studies (10, 11) defines a problematic study as "any published or unpublished study where there are serious questions about the trustworthiness of the data or findings, regardless of whether the study has been formally retracted." We adopt this as a working definition at the outset of the INSPECT-SR project, noting that the project involves the identification of criteria for evaluating 'trustworthiness'. Criteria under consideration could include statistical checks of data and results, aspects of research governance such as ethical approval, presence of plagiarised content, plausibility of the study conduct, or the track record of the research team. Criteria relating to internal or external validity of results produced by RCTs, such as those included in Risk of Bias(12), Risk of Bias 2(13), or GRADE(14) frameworks, are not within the scope of the INSPECT-SR project. The INSPECT-SR tool will be designed to be used alongside these established critical appraisal frameworks. Assessment of

For peer review only

conflicts of interest will be covered by TACIT (Tool for Addressing Conflicts of Interest in Trials)(15) and will not be covered by INSPECT-SR.

INSPECT-SR working group

The INSPECT-SR working group comprises a steering group, an expert advisory panel, a Delphi panel, and additional collaborators. The steering group includes experts in research integrity, clinical trials methodology, systematic reviews, consensus methodology, and methodological guideline development. They will coordinate the development and evaluation of INSPECT-SR. A larger expert advisory panel has been established to provide advice and to contribute throughout the project. This expert panel has been selected to represent a diverse range of relevant expertise and experience. This includes methodologists, research integrity specialists, public contributors, researchers with experience of investigating potentially problematic studies, experts in systematic reviews, and journal editors. Members of the steering group and expert advisory panel were involved as participants in the Stage 1 survey, and may be eligible to participate in the Stage 3 Delphi survey and consensus meeting. Additional collaborators are involved in Stage 2, and may be eligible to participate in the Stage 3 Delphi survey.

Stage 1: Survey of experts to assemble an extensive list of checks

Overall design

The Stage 1 survey of experts has been completed at the time of writing, and a short protocol for the survey has been posted online (https://osf.io/6pmx5/). We describe the methods briefly here. The aim of Stage 1 was to create an extensive list of checks for identifying problematic research studies, which could be taken forward for evaluation in stages 2 and 3. In Stage 1, we did not restrict our focus to checks applicable to or designed for RCTs specifically. Instead, we sought to identify checks applicable to any research design, so that these could be subsequently evaluated for their applicability to RCTs.

We assembled an initial list of 102 checks that could be used to assess potentially problematic studies. The initial list included checks identified in a recent scoping review(16), a recent qualitative study of experts(17) and additional methods known to the research team (for example, JW undertakes integrity investigations for scientific journals and publishers, and added checks known to him as a result of this work). The list was grouped into several preliminary domains, as shown in Table 1 (adapted from https://osf.io/6pmx5/).

We incorporated the list in an online survey in Qualtrics (available at https://osf.io/6pmx5/) to identify checks which had not already been included on the list, and to allow respondents to comment on the checks which were on the list.

For peer review only

The survey asked participants about their experience in assessing potentially problematic studies and to state the country in which they primarily work, before presenting them with the initial list of 102 checks. Each item was presented alongside a free-text box, and participants were advised to comment on any aspect if they wished to do so. At the end of the list, participants were asked whether they were aware of any other checks that had not featured on the list and were presented with a free text box to describe these.

Table 1. Frequency of items grouped into preliminary domains included in an online survey of experts (Stage 1)

Preliminary domain	Frequency of items per domain
1. Inspecting results in the paper	26
2. Inspecting the research team	17
3. Inspecting conduct, governance and transparency	19
4. Inspecting text and publication details	7
5. Inspecting individual participant data	33
	102

Participants and recruitment

People with expertise or experience in assessing potentially problematic studies, before or after publication, were eligible to participate in the survey. We identified eligible individuals primarily through professional networks, including promotion of the project via conference presentations, and by social media. Members of the steering group and expert advisory panel were invited to

For peer review only - http://bmjopen.bmj.com/site/about/guidelines.xhtml

participate. We invited eligible individuals by personalised email, and asked whether they could suggest any other potential participants. We attempted to achieve global representation by monitoring the countries in which the respondents worked as responses accumulated and renewing our efforts to identify and recruit respondents from underrepresented regions. We targeted a minimum sample size of 50, but obtained as many responses as possible.

Analysis and next steps

Descriptive analysis of the survey participants (country of work, experience with assessing potentially problematic studies) and responses will be performed. Additional items suggested by respondents, and comments made on existing items, will be summarised. Based on the survey responses further items will be added to the list, and the wording of existing items will be amended, subject to review by the steering group and expert advisory panel members. The updated list will be taken forward to Stages 2 and 3.

Checks categorised in Domain 5 (Inspecting individual participant data, see Table 1) may only be performed when the underlying dataset for an RCT can be obtained. An extension to the INSPECT-SR tool containing Domain 5 checks is in development (working name *INSPECT-IPD*). The development of INSPECT-IPD requires a different approach to the main INSPECT-SR tool (application of checks to a large sample of individual participant datasets, and a distinct Delphi panel). The remainder of this protocol describes the development of the INSPECT-SR tool, which will include checks in Domains 1 to 4 only.

<u>Stage 2: Retrospective application of the list of items to systematic reviews</u>

Overall design

We will apply the full list of checks we have identified to RCTs in a large sample of systematic reviews of interventions published in the Cochrane library, in order to evaluate their feasibility and impact.

Review selection

We will use a sample of 50 Cochrane Reviews. This sample size has been selected on a pragmatic basis, to allow a sufficient number of applications of the checks to evaluate feasibility and to characterise the impact on results, while remaining achievable. Stage 2 will be undertaken as a large collaborative enterprise, with steering group members, expert advisory panel members, and additional collaborators who have expressed an interest in participating, each applying the full list of checks to the RCTs in a small number of Cochrane Reviews.

We will endeavour to match assessors to topic areas with which they have familiarity, as this reflects how the final INSPECT-SR tool would be used. We will ask each assessor to state a broad topic area relating to their expertise. We will then identify the most recent Cochrane Review relating to this topic and meeting the eligibility requirements. Where an assessor does not have a

For peer review only

particular topic of interest, we will select a topic in order to achieve broad coverage of subjects, and we will identify the most recently published Cochrane Review meeting the eligibility requirements. To be eligible, a Review cannot be authored or co-authored by the assessor, out of concern that this could introduce an incentive to overlook problematic features of included studies. Similarly, the Review should not contain RCTs authored or coauthored by the assessor. The Review must also contain at least one meta-analysis containing between one and five RCTs as a feasibility constraint. We also require that the Review has not undergone an assessment to identify potentially problematic studies already, as this may have resulted in removal of problematic trials from the meta-analyses. We acknowledge however that this final criterion may frequently be unclear.

Data capture

A bespoke data capture form has been produced. Assessors will extract data for each RCT contained in the first meta-analysis in the Cochrane Review which includes between 1 and 5 RCTs. Assessors will initially record their level of familiarity with the topic of the Review (little or no familiarity, some familiarity, high familiarity), and basic information about each RCT, including a study ID based on the names of the first authors of the Review and of the trial, and years of publication of both, and the year of publication of the RCT. Assessors will then extract data for that RCT from the meta-analysis, including sample size per treatment arm and outcome data per treatment arm (e.g. mean and standard deviation for each treatment arm for continuous outcomes, and frequency of events for binary outcomes). The Risk of Bias assessments for that RCT from the Review will be extracted for each domain, as will the corresponding GRADE assessment for the meta-analysis (if there is one).

Assessors will then attempt to apply items from the list of checks from Stage 1 to the RCT. Assessors will be given the opportunity to apply each check, with the exception of checks which require authors of the RCT to be contacted. For each check, assessors will select a response from the options “not feasible”, “passed”, “possibly fail” or “fail”. A free text box will be available for each check so that the assessor may record the reason for their assessment. Finally, having worked through the list of checks, the assessor will record whether they have concerns about the authenticity of the RCT (with options “no”, “some concerns”, “serious concerns”, or “don’t know”), whether they performed any additional checks not included in the list (and if so, what these checks were and what the outcomes were), as well as being given the opportunity to make any additional comments and to estimate how long it took to perform the assessment.

To assist with applying the checks, each assessor will be provided with a guidance document briefly explaining the rationale for each check and instructions on how to apply them. An Excel workbook will be supplied, which can be used to perform some of the statistical checks.

Statistical analysis

We will calculate the frequency of each response option for each check (how often each was considered infeasible, how often each one was failed, possibly failed or passed). We will summarise the overall RCT-level assessments of the assessors after applying the checks

For peer review only

(whether or not they had concerns about authenticity). We will evaluate the impact of removing trials flagged by each item, by comparing the data included in the primary meta-analysis before and after the application of the method (e.g. numbers of trials, numbers of events, sample size) as well as the results (changes in pooled estimate, confidence interval width, heterogeneity). We will visualise the clustering of checks, by plotting trial-level assessments for each check in an array. We will consider the relationship between the assessments and the risk of bias (for each domain) in the reviews, to understand the relationship between indicators of problems on the one hand and assessments of evidence quality on the other. This will be undertaken using multinomial regression to assess the association between assessment and risk of bias ratings for each risk of bias domain. GRADE assessments refer to collections of trials rather than individual trials, and so we will use ordinal regression to assess the association between the number of trials in the meta-analysis flagged and the GRADE rating.

<u>Stage 3: Delphi survey</u>

Overall design

A two-round Delphi survey will be conducted to determine which checks are supported by expert consensus.

Participants and recruitment

Delphi participants will be identified through professional networks of the steering group and expert advisory panel. We will also invite eligible individuals identified and involved in previous stages of the project. We will recruit individuals representing key stakeholder groups, including: individuals with experience or expertise in assessing problematic studies, journal editors, research integrity specialists, systematic reviewers, clinical trialists, and methodologists. We will categorise participants into two larger groups: 1) individuals with expertise or experience in assessing potentially problematic studies and 2) potential users of a tool for assessing potentially problematic studies, noting that participants may be included in both categories. Individuals will be invited via personalised email describing the Delphi survey in the context of the wider INSPECT-SR development project. We will monitor recruitment across stakeholder groups and geographical location and will attempt to improve recruitment for groups in which recruitment numbers are low by targeting potential participants in these groups. We consider at least 30 expert participants in each of the two participant groups (experts and potential users) to represent the minimum for a credible Delphi. However, ideally, we will aim for a minimum of 100 participants overall.

Selection of items

The list of items obtained from Stage 1 will be entered into the Delphi survey. Checks will be categorised and presented in several domains (see Table 1 for the preliminary categorisation scheme, used in Stage 1 but subject to change as the project evolves). We will develop suitable language to clearly describe the checks. The list will be approved by the expert advisory panel

For peer review only

before we launch the Delphi survey, including review by public contributors to confirm clarity. We will write an explanation to accompany each item, which participants may review if they are unsure of its meaning.

Round 1

We will send participants a personalised email outlining the project, together with a link to the survey, which will be implemented online using suitable software. The survey will include the list of checks. In Round 1, respondents will be asked for basic demographic information, to allow categorisation based on domain(s) of expertise. Respondents will be asked to score each check 1 (lowest score) to 9 (highest score) in two dimensions: usefulness and feasibility. Usefulness will relate to the potential effectiveness of the check for detecting a problematic study. Feasibility will relate to the perceived ease of implementation of each check. Participants will also be given the option to indicate that they do not know whether a check is useful or feasible (because, for example, they are unfamiliar with the approach or lack expertise to comment on a particular check). A free-text box will be provided with each check, so that participants may leave any general comments (such as an explanation for their assessment, or suggestions to modify the wording). Round 1 participants will be invited to suggest additional checks.

Round 2

In Round 2, we will add any suggested additional checks to the list (subject to review by the steering group and expert advisory panel), and for each item respondents will be presented with both their own scores (1 to 9) and the distribution of scores from the previous round. Participants who were invited to participate in Round 1 but who did not respond will be invited to the Round 2 survey and will be presented with the distribution of scores from the previous round only. Participants will be asked to provide a new score in light of this information. The Round 2 survey will include a free-text box for each check so that participants may elaborate on their responses.

Analysis

Check-specific scores from Round 2 will be summarised for the overall Delphi panel and by stakeholder group. Any items that meet a consensus criterion, defined as scoring 7 or more by at least 80% of participants overall or in one or more stakeholder groups for usefulness, will be automatically considered during the Stage 4 consensus meeting. Items failing to meet a consensus criterion will be discussed by the steering group and expert advisory panel in light of the Stage 2 application exercise and will be considered for inclusion in the Stage 4 consensus meetings. Feasibility scores will be summarised for each check and will be used in Stage 4.

<u>Stage 4: Consensus meetings</u>

Consensus meetings will be held to finalise the checks to be included in the draft INSPECT-SR tool. We anticipate that multiple meetings will be necessary in order to accommodate international time differences. Meetings may be virtual, in-person, or a combination of both. At these meetings,

For peer review only

the results of the Stage 2 application exercise and Stage 3 Delphi survey will be discussed, with the purpose of finalising the items to be included in the draft INSPECT-SR tool. The feasibility assessments from the Stage 2 application exercise and Stage 3 Delphi survey will be considered for all items discussed. Items that are considered useful but challenging to implement may not be incorporated into the main tool, but instead included as an optional or recommended check in the accompanying guidance document. Participants will be invited to reflect the range of key stakeholder groups, as described above. We anticipate that 20 to 30 participants will participate in the consensus meetings, with ten to fifteen participants representing each of the two main participant groups (experts and potential users). In addition to determining the checks to include in the tool, it will be necessary to determine its form and structure, and the recommended process for applying it during the systematic review process. It may be necessary to hold additional meetings focussed on these questions.

Stage 5: Prospective testing of draft tool

Overall design

In collaboration with systematic reviewers, we will prospectively evaluate the draft tool by using it in the production of a cohort of new systematic reviews and systematic review updates. The impact of the draft tool's Impact on Review conclusions will be assessed in the same way as in Stage 2. We will assess feasibility and usability by implementing surveys regarding experiences of use. Separate surveys will be designed for review authors and, for Cochrane Reviews, editors. These will explore ease of implementation, barriers to use, and suggestions for improvement. In addition to user-level data, we will capture data relating to the individual reviews in which the tool was implemented, as each one represents a potentially informative case study. We will undertake additional qualitative interviews with users during this testing phase, to capture additional feedback.

We will aim to include a variety of topic areas in this testing phase. Stage 5 will culminate in a user workshop, including review editors and review authors involved in testing the tool.

User workshop

Findings from the surveys will be fed back to participants as part of a user workshop. The workshop might be virtual, in-person, or a combination of both. Participants will share their experiences of using the tool, and make recommendations for refinement. The discursive format of the meeting is intended to reveal additional information about the experience of users that could not be easily captured via the surveys. We will invite both authors and editors involved in the testing phase to participate. The findings of the testing phase will be used to make final modifications to the tool for usability. We will use the results to produce guidance relating to use of the tool in practice. Alongside Stage 5, as we gather user data, we will produce training materials (to be delivered as workshops and as an online training module) to familiarise

For peer review only

systematic review authors and editors with the tool. These will be finalised in light of the findings from the user workshop.

Patient and public involvement

An outline for the project was reviewed and commented by patient partners prior to grant submission. The expert advisory panel includes two lay members, who are given equal opportunity to contribute to the design and dissemination of all work packages. One lay member is acting as a co-author on the current protocol manuscript.

ETHICS AND DISSEMINATION

The University of Manchester ethics decision tool was used, and this returned the result that ethical approval was not required for this project (30th September 2022), which incorporates secondary research and surveys of professionals about subjects relating to their expertise. Informed consent will be obtained from all survey participants. All results will be published as open-access articles. The final tool will be freely available.

DISCUSSION

Systematic reviews of health interventions are considered to represent a very high standard of evidence and frequently inform policy and practice. However, because the veracity of included RCTs is not usually considered, systematic reviews may unintentionally act as a pipeline for false data with the risk that this will influence care. While the need to prevent problematic studies from contributing to systematic reviews is recognised, with several recent laudable efforts to tackle the issue (18-20), there is currently limited agreement on how this should be done. The INSPECT-SR project will develop a tool for evaluating the trustworthiness studies, backed by empirical evidence and expert consensus.

The topic of trustworthiness is understandably contentious. To be credible, a tool for assessing trustworthiness should have broad backing from the health research community. For this reason, a large, international consensus process will inform the development of the INSPECT-SR tool. The project will involve key stakeholders, including people with expertise in designing, developing and publishing RCTs and systematic reviews, as well as patient partners. We anticipate that this inclusive approach will aid with dissemination of the tool, and training materials will be produced to facilitate effective use. There is no gold standard test for inauthentic data, and accordingly, INSPECT-SR is not being designed as a diagnostic test for fraud. Rather, we anticipate that the tool will guide the user through a series of checks to help them make a judgement about the trustworthiness of a study.

We anticipate the draft tool will be available early 2024, and the final tool will be available late 2024.

Funding

For peer review only

This research is funded by the NIHR Research for Patient Benefit programme (NIHR203568). The views expressed are those of the author(s) and not necessarily those of the NIHR or the Department of Health and Social Care.

Competing interests

JW, CH, GA, LB, JJK declare funding from NIHR (NIHR203568) in relation to this work. JW additionally declares Stats or Methodological Editor roles for BJOG, Fertility and Sterility, Reproduction and Fertility, Journal of Hypertension, and for Cochrane Gynaecology and Fertility. CH declares a Statistical Editor role for Cochrane Colorectal. LB additionally declares a role as Academic Meta-Research Editor for PLoS Biology, and that The University of Colorado receives remuneration for service as Senior Research Integrity Editor, Cochrane. JJK additionally declares a Statistical Editor role for The BMJ. WL, ALS, and RW declare funding from Australian National Health and Medical Research Council Investigator Grants (GNT2016729, GNT2009432, GNT2009767). ALS additionally declares roles as Editorial Board member for Cochrane Evidence Synthesis Methods and Statistical Editor for Cochrane Neonatal Group. RW additionally declares roles as Deputy Editor for Human Reproduction and Editorial Board Member for BJOG and Cochrane Gynaecology and Fertility. WL additionally declares roles of Associate Editor for Human Reproduction Open and Methodolical Editor for Fertility and Sterility and Fertility and Sterility Reviews. EF, SG, TLa declare employment by Cochrane. EF additionally declares a role as Editorial Board member for Cochrane Synthesis and Methods. TLa additionally declares authorship of a chapter in the Cochrane Handbook for Systematic Reviews of Interventions and that he is a developer of standards for Cochrane intervention reviews (MECIR). SG additionally declares an Academic Editor role for PLOS Global Public Health. ES declares that she was a member of the Cochrane scientific misconduct policy advisory group, and roles as Sign-off Editor and Proposal Editor of The Cochrane Library. ZA declares he is a member of the Cochrane Library Editorial Board, was Co-Ordinating Editor for Cochrane Pregnancy and Childbirth Group 2002-2023, and is PI on a grant from Children Investment Foundation Fund to University of Liverpool to investigate research integrity of clinical trials related to nutritional supplements in pregnancy. TLi is supported by grant UG1 EY020522 from the National Eye Institute, National Institutes of Health. Tli also declares roles as Editor-in-Chief for Trials, Statistical Editor for Annals of Internal Medicine, Review Editor for JAMA Opthalmology, and Editorial Board member for Journal of Clinical Epidemiology. MC declares that he is Co-ordinating Editor for the Cochrane Methodology Review Group. AA declares that The Health Services Research Unit, University of Aberdeen, is funded by the Health and Social Care Directorates of the Scottish Government. All other authors declare no competing interests. DT declares an RCT Topic Editor role for Research Methods in Medicine and Health Sciences. MvW declares roles as Co-ordinating Editor for Cochrane Gynaecology and Fertility and Sexually Transmitted Infections, Methodology Editor for Human Reproduction Update, and Editorial Editor for Fertility and Sterility. NEOC declares roles as Co-ordinating Editor for Cochrane Pain, Palliative and Supportive Care (PaPaS) Group, 2020-2023, as Member of Cochrane Central Editorial Board, and as Editorial Board member for Journal of Pain. JC declares an Editor role for Anaesthesia. AL declares a role as Editorial Board member for BMC Medical Ethics. GAA declares a role as Statistical Editor for the European Journal of Vascular and Endovascular Surgery. NJLB declares roles as Editorial Board member for International Review of Social Pyschology/ Revue Internationale de Psychologie Sociale,

For peer review only

Statistical Advisory Board member for Mental Health Science, and Advisory Board member for Meta-Psychology. BWM declares roles as Editor for Cochrane Gynaecology and Fertility and Sexually Transmitted Infections and for Fertility and Sterility. SL declares roles as Associate Editor for Human Reproduction, Methodological Editor for Fertility and Sterility, and Editor for Cochrane Gynaecology and Fertility. EL is Head of Research and Clinical Editor for The BMJ.

Contributors

JW, LB, JJK, GAA developed the initial idea for the project and obtained the funding to complete the work. JW, CH, GAA, EF, JJK, LB form the project Steering Group. JW drafted the initial version of the manuscript. JW, CH, GAA, EF, JJK, LB, ZA, AA, VB, NJLB, JC, MC, PD, JD, AG, SG, LCG, JAH, JH, KEH, TLa, EL, SL, TLi, WL, EL, AL, GMK, BWM, NEOC, LP, BKR, ALS, KAS, ES, DJT, MVW, RW are members of the project Expert Panel, developed the protocol, co-authored the manuscript and approved the submitted version. Data analysis will be conducted by CH and JW. JW will draft initial versions of manuscripts arising from the project.

For peer review only

References

1. Bryant A, Lawrie TA, Fordham EJ. Ivermectin for Prevention and Treatment of COVID-19 Infection: A Systematic Review, Meta-analysis, and Trial Sequential Analysis to Inform Clinical Guidelines. American Journal of Therapeutics, 28, e434-e460, July 2021. Am J Ther. 2021;28(5):e573-e6.
2. Hill A, Garratt A, Levi J, Falconer J, Ellis L, McCann K, et al. Meta-analysis of Randomized Trials of Ivermectin to Treat SARS-CoV-2 Infection. Open Forum Infect Dis. 2021;8(11):ofab358.
3. Lawrence JM, Meyerowitz-Katz G, Heathers JAJ, Brown NJL, Sheldrick KA. The lesson of ivermectin: meta-analyses based on summary data alone are inherently unreliable. Nat Med. 2021;27(11):1853-4.
4. Brown NJL. http://steamtraen.blogspot.com/2021. [cited 2023]. Available from: http://steamtraen.blogspot.com/2021/07/Some-problems-with-the-data-from-a-Covid-study.html.
5. Hill A, Mirchandani M, Pilkington V. Ivermectin for COVID-19: Addressing Potential Bias and Medical Fraud. Open Forum Infect Dis. 2022;9(2):ofab645.
6. Cockayne S, Adamson J, Lanham-New S, Shearer MJ, Gilbody S, Torgerson DJ. Vitamin K and the prevention of fractures: systematic review and meta-analysis of randomized controlled trials. Arch Intern Med. 2006;166(12):1256-61.
7. Grey A, Avenell A, Bolland M. Revised Meta-analysis of Vitamin K and Fractures. JAMA Intern Med. 2018;178(8):1135.
8. Ker K, Shakur H, Roberts I. Does tranexamic acid prevent postpartum haemorrhage? A systematic review of randomised controlled trials. BJOG. 2016;123(11):1745-52.
9. Williams ACC, Fisher E, Hearn L, Eccleston C. Psychological therapies for the management of chronic pain (excluding headache) in adults. Cochrane Database Syst Rev. 2020;8(8):CD007407.
10. Cochrane. Cochrane Database of Systematic Reviews: editorial policies Cochrane Library [Available from: https://www.cochranelibrary.com/cdsr/editorial-policies.

11. Boughton SL, Wilkinson J, Bero L. When beauty is but skin deep: dealing with problematic studies in systematic reviews. Cochrane Database Syst Rev. 2021;6(6):ED000152.
12. Higgins JP, Altman DG, Gotzsche PC, Juni P, Moher D, Oxman AD, et al. The Cochrane Collaboration's tool for assessing risk of bias in randomised trials. BMJ. 2011;343:d5928.
13. Sterne JAC, Savovic J, Page MJ, Elbers RG, Blencowe NS, Boutron I, et al. RoB 2: a revised tool for assessing risk of bias in randomised trials. BMJ. 2019;366:l4898.
14. Guyatt GH, Oxman AD, Kunz R, Vist GE, Falck-Ytter Y, Schünemann HJ. What is "quality of evidence" and why is it important to clinicians? BMJ. 2008;336(7651):995-8.
15. Tool for Addressing Conflicts of Interest in Trials [Available from: https://tacit.one/.
16. Bordewijk EM, Li W, van Eekelen R, Wang R, Showell M, Mol BW, et al. Methods to assess research misconduct in health-related research: A scoping review. J Clin Epidemiol. 2021;136:189-202.
17. Parker L, Boughton S, Lawrence R, Bero L. Experts identified warning signs of fraudulent research: a qualitative study to inform a screening tool. J Clin Epidemiol. 2022;151:1-
18. Mol BW, Lai S, Rahim A, Bordewijk EM, Wang R, van Eekelen R, et al. Checklist to assess Trustworthiness in RAndomised Controlled Trials (TRACT checklist): concept proposal and pilot. Res Integr Peer Rev. 2023;8(1):6.
19. Weibel S, Popp M, Reis S, Skoetz N, Garner P, Sydenham E. Identifying and managing problematic trials: A research integrity assessment tool for randomized controlled trials in evidence synthesis. Res Synth Methods. 2023;14(3):357-69.
20. Grey A, Bolland MJ, Avenell A, Klein AA, Gunsalus CK. Check for publication integrity before misconduct. Nature. 2020;577(7789):167-9.

Figure title:

Figure 1. INSPECT-SR development process

For peer review only

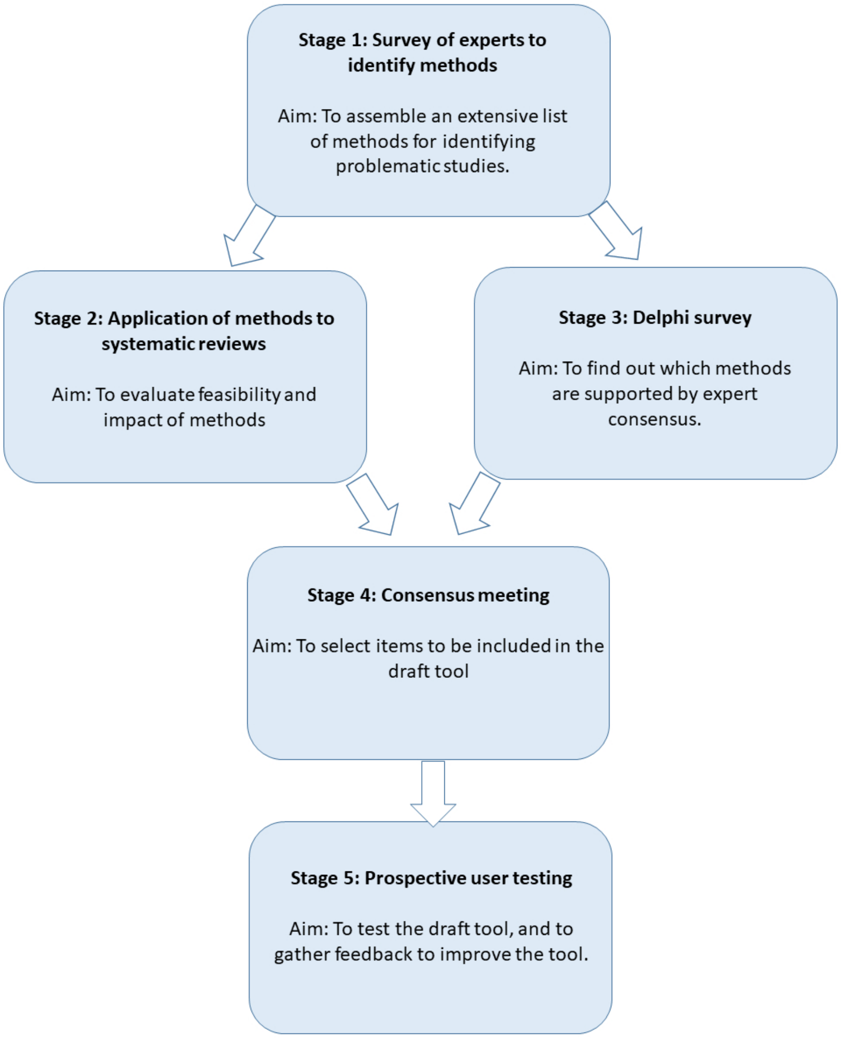

INSPECT-SR development process

302x338mm (96 x 96 DPI)
