# Supplementary material for: Protocol for the development of a tool (INSPECT-SR) to identify problematic randomised controlled trials in systematic reviews of health interventions
Source: BMJ Open. 2024 Mar 11;14(3):e084164. doi: 10.1136/bmjopen-2024-084164 (PMC10936473; doi:10.1136/bmjopen-2024-084164)
Supplement: Reviewer comments [file bmjopen-2024-084164.reviewer_comments.pdf]

PEER REVIEW HISTORY

BMJ Open publishes all reviews undertaken for accepted manuscripts. Reviewers are asked to complete a checklist review form (**http://bmjopen.bmj.com/site/about/resources/checklist.pdf**) and are provided with free text boxes to elaborate on their assessment. These free text comments are reproduced below.

ARTICLE DETAILS

**TITLE (PROVISIONAL)**	Protocol for the development of a tool (INSPECT-SR) to identify problematic randomised controlled trials in systematic reviews of health interventions
**AUTHORS**	Wilkinson, Jack; Heal, Calvin; Antoniou, George; Flemyng, Ella; Alfirevic, Zarko; Avenell, Alison; Barbour, Ginny; Brown, Nicholas; Carlisle, John; Clarke, Mike; Dicker, Patrick; Dumville, Jo C.; Grey, Andrew; Grohmann, Steph; Gurrin, Lyle; Hayden, Jill; Heathers, James; Hunter, Kylie; Lasserson, Toby; Lam, Emily; Lensen, Sarah; Li, Tianjing; Li, Wentao; Loder, Elizabeth; Lundh, Andreas; Meyerowitz-Katz, Gideon; Mol, Ben; O'Connell, Neil; Parker, Lisa; Redman, Barbara; Seidler, Lene; Sheldrick, Kyle; Sydenham, Emma; Torgerson, David; van Wely, MadelonEditorial Board Member; Wang, Rui; Bero, Lisa; Kirkham, Jamie J.

This article was not externally reviewed at BMJ Open. Protocols that have been independently assessed prior to submission to BMJ Open are usually fast-tracked to publication on the grounds that further substantial changes will not be possible. This independent assessment will usually be external, independent review for both a substantial grant award from a non-commercial or government funder and ethics approval.
